# Necrotizing Fasciitis

**DOI:** 10.1155/S1064744996000403

**Published:** 1996

**Authors:** David E. Soper

**Affiliations:** Medical University of South Carolina 171 Ashley Avenue Charleston SC 29401-2233 USA


					Infectious Diseases in Obstetrics and Gynecology 4:214 (1996)
(C) 1997 Wiley-Liss, Inc.
Images in Infectious Diseases in Obstetrics and Gynecology
Section Editor: David E. Soper, M.D.
Necrotizing Fasciitis
David E. Soper, M.D.
Medical University of South Carolina
Charleston, SC 29401
LEGEND TO IMAGE
Necrotizing fasciitis is an uncommon but life-threatening
infection primarily involving the superficial fascia. Note
the blue-gray color change of the vulvar skin lateral to
areas of ulceration (A), The superficial fascia ofthe vulva
is in continuity with that of the anterior abdominal wall.
This incision reveals normal-appearing subcutaneous tis-
sue in its superior aspect compared to the dull gray-
appearing necrotic tissue inferiorly (B). A portion of the
resected specimen shows a line of demarcation between
the healthy yellow vascular subcutaneous tissue on the
right and the necrotic tissue on the left (C). Surgical
resection of the superficial fascia should include these
margins in order to successfully treat the disease.
(C) 1996 Wiley-Liss, Inc.
A
B
Images in Infectious Diseases in Obstetrics and Gynecology presents clinically important visual images that a practitioner in
women's health might encounter. If you have a high-quality color or black-and-white photograph or slide representing such
an image that you would like considered for publication, send it with a descriptive legend to David E. Soper, MD, Medical
University of South Carolina, 171 Ashley Avenue, Charleston, SC 29401-2233.
Images is made possible through an educational grant from Pfizer, Inc.
Images
Received June 19, 1996
Accepted June 19, 1996

				

